# Analgesic effect of Dahuang Fuzi Decoction in neuropathic pain through inhibiting TNF-α and PI3K-AKT signaling

**DOI:** 10.3389/fnins.2024.1464477

**Published:** 2024-12-11

**Authors:** Jinglian Qu, Qian Gong, Siyu He, Jiuyan Peng, Lingyan Chen, Long Wang, Peng Chen

**Affiliations:** ^1^Basic Medical School, Guizhou University of Traditional Chinese Medicine, Guiyang, Guizhou, China; ^2^First Clinical Medical School, Guangzhou University of Chinese Medicine, Guangzhou, Guangdong, China; ^3^School of Pharmacy, Southwest Medical University, Luzhou, Sichuan, China; ^4^Department of Rehabilitation, The First Affiliated Hospital of Guangzhou Medical University, Guangzhou, China

**Keywords:** Dahuang Fuzi Decoction, neuropathic pain, TNF signaling pathway, network pharmacology, PI3K-AKT signaling

## Abstract

**Background:**

Neuropathic pain (NeP) presents considerable challenges in terms of effective management and significantly impacts the quality of life for affected patients. The current treatment options for NeP are limited, highlighting the need for alternative therapeutic approaches. Dahuang Fuzi Decoction (DF), a formula from traditional Chinese medicine, has shown potential in relieving pain symptoms associated with various types of NeP. However, the mechanisms through which DF exerts its effects remain largely unknown.

**Methods:**

In this study, we employed ultra-high-performance liquid chromatography coupled with high-resolution mass spectrometry (UHPLC-HRMS) to analyze the chemical composition of DF. A chronic sciatic nerve compression injury (CCI) rat mode was used to assess the analgesic efficacy of DF for NeP. Network pharmacology analysis was performed to identify the potential signaling pathways affected by DF.

**Results:**

DF treatment significantly increased the mechanical withdrawal threshold (MWT) and thermal withdrawal latency (TWL) in CCI rats, indicating its analgesic effect. Network pharmacology analysis suggested that DF potentially modulated TNF-α and PI3K-AKT signaling pathways. Furthermore, DF treatment decreased the levels of pro-inflammatory cytokines (IL-1β, IL-6, and TNF-α) in spinal cord tissues of CCI rats, suggesting an anti-inflammatory effect. Western blot analysis revealed that DF treatment reduced the expression of TNF-α, TNFR1, and phosphorylated forms of PI3K, AKT, IKKα/β, IKBα, and NF-κB in the spinal cord of CCI rats. Immunofluorescence analysis confirmed significant reductions in TNF-α and TNFR1 expression, as well as in AKT and NF-κB phosphorylation within astrocytes following DF administration.

**Conclusion:**

Our findings characterize the chemical constituents of DF and elucidate its underlying mechanism for relieving NeP. The analgesic effect of DF involves the inhibition of TNF-α and PI3K-AKT signaling pathways, providing a potential therapeutic approach for NeP management.

## 1 Introduction

Neuropathic pain (NeP) is a form of chronic pain arising from injury or disease affecting the somatosensory nervous system, which may be caused by factors such as surgical incisions, nerve compression, autoimmune disorders, or channelopathies. NeP affects ~7–10% of the population and imposes a substantial burden on patients (Racine et al., 2016). Current first-line treatments for NeP consist of tricyclic antidepressants (TCAs), serotonin-noradrenaline reuptake inhibitors, pregabalin, and gabapentin (Dosenovic et al., 2017). Although these medications target different mechanisms involved in NeP progression, their effectiveness is often limited, and they can be associated with severe side effects (Finnerup et al., 2015, 2021). Therefore, it is crucial to develop new therapeutic approaches to better manage NeP.

The spinal cord serves as the central hub for integrating and transmitting nociceptive signals (Wang et al., 2017). Central sensitization, marked by increased neuronal and circuit activity within the spinal cord, plays a crucial role in the development and maintenance of NeP (Latremoliere and Woolf, 2009; Lutolf et al., 2022). Microglia, the resident immune cells of the central nervous system, are key contributors to this process, with microglia-mediated neuroinflammation being a significant factor driving central sensitization in NeP (Inoue and Tsuda, 2018; Ji et al., 2018). Following nerve damage, microglia become rapidly activated, undergoing morphological changes, microgliosis, and increased transcriptional activity (Inoue and Tsuda, 2018). The activated cells subsequently release proinflammatory mediators such as tumor necrosis factor-α (TNF-α), IL-1β, and brain-derived neuropathic pain (BDNF), which influence synaptic activity across spinal segments, promoting central sensitization (Popiolek-Barczyk and Mika, 2016; Zhao et al., 2017; Ji and Xu, 2021). Consequently, targeting microglia-induced inflammation represents a promising therapeutic strategy for managing NeP (Fiore et al., 2023; Wang et al., 2023).

Recent studies have demonstrated the notable analgesic effects of traditional Chinese medicine (TCM) in managing NeP, both in clinical settings and animal models (Liu et al., 2021, 2022). Dahuang Fuzi Decoction (DF), a classical TCM formulation first documented in the *Synopsis of Prescriptions of the Golden Chamber*, comprises Rhubarb, Radix Aconiti Lateralis, and Asarum heterotropoides. It is commonly used to treat inflammatory conditions, neurological disorders, and headaches (Tai et al., 2021; Gu et al., 2022). Clinically, we have observed that DF can effectively alleviate pain symptoms in patients with various NeP conditions, such as sciatica and post-herpetic neuralgia. Despite these promising effects, the mechanisms underlying DF's analgesic properties in NeP remain poorly understood. This study aims to investigate the effects of DF in a chronic constriction injury (CCI) model and explore its mechanisms of action through network pharmacology and experimental validation.

## 2 Materials and methods

### 2.1 Preparation of DF

DF herbal granules, consisting of Rhubarb (Batch No. A2092591), Radix Aconiti Lateralis (Batch No. A2071291), and Asarum heterotropoides (Batch No. A209A882), were sourced from the Guangdong Yifang Chinese Herbal Medicine Department in a 3:4:1 ratio.

### 2.2 Fingerprint analysis of DF

Fingerprint analysis of DF was perfomed using a Thermo Vanquish Flex Ultra Performance Liquid chromatography system coupled with a Thermo Fisher QE high-resolution mass spectrometer (ThermoFisher, USA). Separation of the DF constituents was achieved with a Thermo Scientific HyPURITY C18 column (150 × 2.1 mm, 1.6 μm). The mobile phase consisted of methanol (solvent A) and 0.1% (v/v) phosphoric acid (solvent B), with the following elution gradient: 0–5 min, 3–21% A; 5–20 min, 21–36% A; 20–32 min, 36–50% A; 32–42 min, 50–62% A; 42–50 min, 62–85% A; 50–60 min, 85–95% A. The injection volume was 1 μL, and the column temperature was maintained at 30°C. Detection was carried out at 260 nm with a flow rate of 0.2 mL/min.

### 2.3 Network pharmacology analysis

#### 2.3.1 Identification of potential targets of DF

The ingredients of DF identified through high-resolution mass spectrometry were screened for oral bioavailability (OB) ≥30% and drug-likeness (DL) ≥0.18 to qualify as active compounds. The targets associated with these active compounds were then obtained from the SwissTargetPrediction database (http://www.swisstargetprediction.ch/). Subsequently, all identified targets were cross-referenced with the UniProt database (https://www.uniprot.org/) to obtain annotated and reviewed gene symbols. Duplicate entries and non-standard targets were excluded to establish the final list of potential DF targets (Shi et al., 2019; Zhang J. Y. et al., 2019; Zhang J. et al., 2019).

#### 2.3.2 Target prediction of DF for treating NeP

NeP-related targets were retrieved from the GeneCards database (http://www.genecards.org/), considering only genes with a relevance score of ≥1. Related targets associated with neuropathic pain were further compiled by integrating data from multiple databases, including DisGeNET (https://www.disgenet.org/) and DrugBank (http://www.drugbank.ca/), among others. By comparing the predicted targets of the active ingredients in DF with the NeP-related targets obtained, overlapping targets were identified as relevant to DF's treatment of NeP. The interactions between compounds and targets were visualized using a bioinformatics platform (www.bioinformatics.com.cn).

#### 2.3.3 Network construction

The targets were processed by Cytoscape 3.7.2 software to construct a representation of the “drug disease–active ingredients–intersection targets.” For protein-protein interaction (PPI) data visualization, the String database (https://string-db.org/) was employed, with the species parameter set to “Homo sapiens.” During this process, only interactions with a *P*-value < 0.05 were retained. The significant potential targets of DF, along with those related to NeP treatment, were subsequently uploaded to Cytoscape 3.7.2 for further analysis.

#### 2.3.4 Enrichment analysis of targets of DF against NeP

The Metascape database (https://metascape.org/) was utilized for conducting Gene Ontology (GO) analysis and Kyoto Encyclopedia of Genes and Genomes (KEGG) pathway enrichment analysis. Initially, the targets of DF against NeP were inputted into the Metascape database, which automatically annotated the gene IDs. Subsequently, the species option was set as “Homo sapiens.” A significance threshold of *P* ≤ 0.05 was chosen as the default option. Finally, the results of the GO analysis and KEGG pathway enrichment analysis were obtained.

### 2.4 Animals

Thirty-six-week-old male Sprague-Dawley (SD) rats (200 ± 20 g) were sourced from Guangdong Medical Laboratory Animal Center (Certificate NO. SCXK[Guangdong]2022-0002, Guangzhou, China). The temperature and relative humidity were maintained at 26°C and 60–70%, respectively, under a 12 h dark-light cycle. The animals were randomly divided into six groups (*n* = 6 per group): sham group, CCI group, low-dose DF group (DF-L), medium-dose DF group (DF-M), high-dose DF group (DF-H), and pregabalin group (PGB). The study adhered to the ethical guidelines and regulations approved by the Animal Experimentation Ethics Committee at The First Affiliated Hospital of Guangzhou University of Chinese Medicine (License No. GZTCMF1-2021100).

### 2.5 CCI surgery and DF treatment

The CCI model was established according to previously published protocols after inducing anesthesia with an intraperitoneal injection of pentobarbital sodium (Chen et al., 2023a,b). The left sciatic nerve was carefully exposed and ligated with four silk ligatures (4-0) at an average interval of 1–2 mm. The incision was closed in layers postoperatively. In the sham group, the sciatic nerve was exposed but not ligated. Rats in the DF-L (2.4 g/kg), DF-M (4.8 g/kg), and DF-H (9.6 g/kg) groups received DF solution via oral gavage once daily for 15 days, starting on the first postoperative day. The positive control group received pregabalin solution (15 mg/kg, Batch No. J20160021, Pfizer).

### 2.6 Behavioral test

#### 2.6.1 Mechanical withdrawal threshold

The mechanical withdrawal threshold (MWT) was assessed using an electronic von Frey anesthesiometer (IITC Life Science Instruments, Woodland Hills, CA, USA; Chen et al., 2023a,b). Rats were placed in a clear Plexiglas chamber for 30 min prior to testing. The anesthesiometer tip was applied to stimulate the ipsilateral mid-plantar area of each rat three times, with a 5-min interval between stimuli. The onset of paw lifting or licking was recorded, and the average value was calculated to determine the MWT.

#### 2.6.2 Thermal withdrawal latency

Thermal withdrawal latency (TWL) was measured using a thermal radiation stimulator (IITC Life Science Instruments, Woodland Hills, CA, USA; Chen et al., 2023a,b). Prior to the test, rats were acclimated in a transparent Plexiglas box for 30 min. The radiant heat source was applied to the ipsilateral mid-plantar area three times, with a 10-min interval between exposures. The appearance of paw lifting or licking was noted, and the average TWL was calculated.

### 2.7 Enzyme-linked immunosorbent assay analysis

After the behavioral tests on day 15 post-surgery, rats were deeply anesthetized with an intraperitoneal injection of pentobarbital (40 mg/kg). Spinal cord tissues were harvested and homogenized using a tissue homogenizer. Levels of inflammatory cytokines (IL-1β, IL-6, and TNF-α) in the spinal cord were quantified using ELISA kits (Meimian, Jiangsu, China) following the manufacturer's instructions.

### 2.8 Western blot analysis

Western blotting was performed to assess the protein expressions of TNF-α, TNFR1, PI3K, AKT, IKKα/β, IKBα, NF-κB, p-PI3K, p-AKT, p-IKKα/β, p-IKBα, and p-NF-κB. Briefly, spinal cord tissues were lysed in RIPA buffer (Meilunbio, Dalian, China) and centrifuged at 12,000 × *g* for 5 min at 4°C. The supernatant was collected after dilution and denaturation. Protein samples of 40 μg were then subjected to sodium dodecyl sulfate-polyacrylamide gel electrophoresis (SDS-PAGE) for separation. Following electrophoresis, proteins were transferred to a polyvinylidene difluoride membrane. After blocking with TBST containing 5% skim milk powder or 1% bovine serum albumin (for phosphorylated proteins) for 2 h, membranes were incubated overnight at 4°C with primary antibodies. The membranes were then incubated with a secondary antibody (IRDye 800CW Goat anti-Rabbit, Boster, Wuhan, China). The primary antibodies used included anti-TNFα (Beyotime, Shanghai, China), anti-TNFR1 (Abclonal, Wuhan, China), anti-PI3K (Affinity, Jiangsu, China), anti-AKT (Affinity, Jiangsu, China), anti-IKKα/β (Affinity, Jiangsu, China), anti-IKBα (Affinity, Jiangsu, China), anti-NF-κB (Affinity, Jiangsu, China), anti-p-PI3K (Affinity, Jiangsu, China), anti-p-AKT (Affinity, Jiangsu, China), anti-p-IKKα/β (Affinity, Jiangsu, China), anti-p-IκBα (Affinity, Jiangsu, China), anti-p-NF-κB (Affinity, Jiangsu, China), and anti-GAPDH (Goodhere, Hangzhou, China). Bands were quantified using Image-Pro Plus 6.0.

### 2.9 Immunofluorescence

Immunofluorescence assays were conducted following established protocols. Briefly, spinal cord tissues were dehydrated with increasing concentrations of alcohol and subsequently cleared with xylene. The cleared tissues were embedded in paraffin, and sections were prepared using a microtome. Paraffin was removed from the sections, and antigen retrieval was performed with an electric heat-retrieval device. Normal serum was applied around the sections for blocking. Diluted primary antibodies were added and incubated overnight. Following washes, sections were treated with Cy3-labeled goat anti-rabbit IgG secondary antibody (Boster, Wuhan, China). Nuclear staining was achieved using DAPI. Excess liquid was removed, and sections were mounted and visualized under a fluorescence microscope. The primary antibodies utilized included anti-TNFR1 (Abclonal, Wuhan, China), anti-TNF-α (Beyotime, Shanghai, China), and anti-p-NF-κB (Affinity, Jiangsu, China).

### 2.10 Molecular docking

For molecular docking, the three-dimensional structure of the target protein was retrieved from the Protein Data Bank (PDB). The protein was then optimized using specialized software such as PyMOL or Chimera. During optimization, water molecules were removed, missing hydrogen atoms were added, and any non-standard residues were corrected to ensure a reliable protein structure. The three-dimensional structure of the ligand was obtained from a relevant database like PubChem, and the ligand was optimized using molecular editing software such as Open Babel or Avogadro. This optimization involved adding hydrogen atoms, adjusting charge states, and generating Gasteiger-Marsili type charges for accurate ligand representation. Finally, the prepared protein and ligand structure files were imported into Autodock Vina for docking simulations.

### 2.11 Statistical analysis

Experimental data are presented as means ± standard deviations and analyzed using one-way analysis of variance (ANOVA) in IBM SPSS Statistics 26.0. A *p*-value of < 0.05 was considered statistically significant.

## 3 Results

### 3.1 Characterization of ingredients of DF

**Figure 1:**
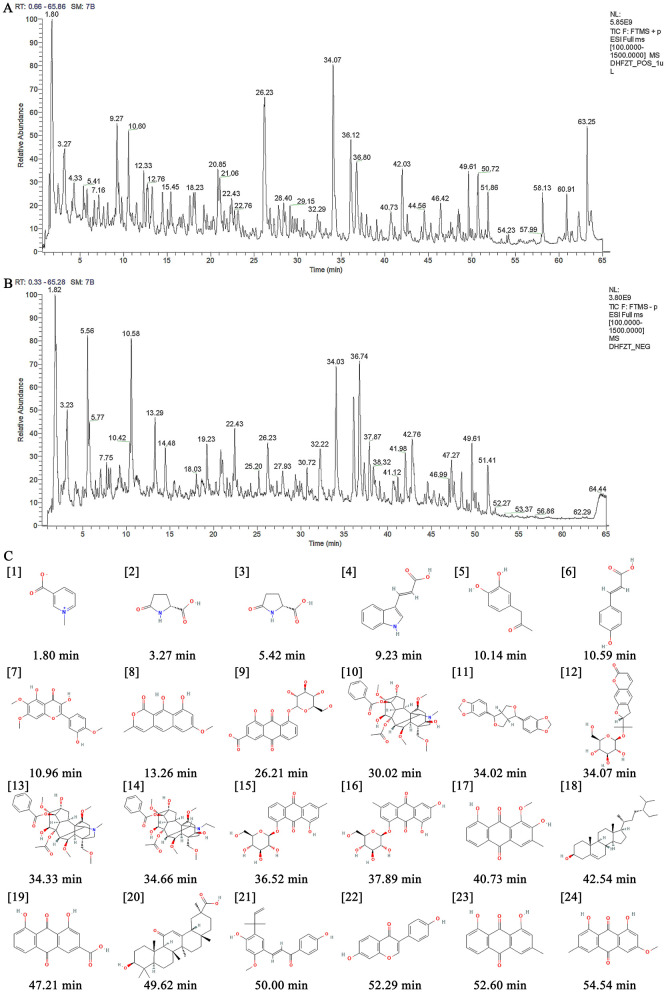
Identification of ingredients of DF using UHPLC-HRMS. **(A)** The total ion current chromatogram of DF under the positive mode. **(B)** The total ion current chromatogram of DF under the negative mode. **(C)** Chemical structures of identified compounds present in DF.

The ingredients of DF were identified using ultra-high-performance liquid chromatography coupled with high-resolution mass spectrometry (UHPLC-HRMS). Figures 1A, B illustrate the total ion current chromatograms, while Table 1 lists the identified components. A total of 24 ingredients were tentatively characterized, with their structural formulas presented in Figure 1C.

**Table 1:** The compounds of aqueous extracts of DF.

**No**.	**Identity**	**Mode**	**Molecular formula**	**RT (min)**	**[M+H]+ (m/z)**
1	Trigonelline	Pos	C_7_H_7_NO_2_	1.80	138.05487
2	D-Pyroglutamic acid	Pos	C_5_H_7_NO_3_	3.27	130.04991
3	Adenosine	Pos	C_10_H_13_N_5_O_4_	5.42	268.10385
4	Trans-3-Indoleacrylic acid	Pos	C_11_H_9_NO_2_	9.23	188.07047
5	3′,4′-Dihydroxyphenylacetone	Pos	C_9_H_10_O_3_	10.14	167.07033
6	4-Coumaricacid	Pos	C_9_H_8_O_3_	10.59	165.05456
7	Eupatin	Pos	C_13_H_12_O_3_	10.96	217.08688
8	Toralactone	Pos	C_15_H_12_O_5_	13.26	273.07541
9	Rhein-8-o-beta-o-glucoside	Neg	C_21_H_18_O_11_	26.21	445.07440
10	Mesaconitine	Pos	C_33_H_45_NO_11_	30.02	632.30652
11	Sesamin	Pos	C_20_H_18_O_6_	34.02	355.11758
12	Nodakenin	Pos	C_20_H_24_O_9_	34.07	431.13037
13	Hypaconitine	Pos	C_33_H_45_NO_10_	34.33	616.31067
14	Aconitine	Pos	C_34_H_47_NO_11_	34.66	646.32214
15	Chrysophanol-1-O-β-D-glucoside	Neg	C_21_H_20_O_9_	36.52	415.10300
16	Emodin-1-O-β-D-glucopyranoside	Pos	C_21_H_20_O_10_	37.89	455.09467
17	Obtusifolin	Neg	C_16_H_12_O_5_	40.73	285.07526
18	Beta-sitosterol	Pos	C_29_H_50_O	42.54	415.13406
19	Rhein	Pos	C_15_H_8_O_6_	47.21	283.06080
20	Glycyrrhetinic acid	Neg	C_30_H_46_O_4_	49.62	453.33557
21	Licochalcone A	Pos	C_21_H_22_O_4_	50.00	361.14142
22	Daidzein	Pos	C_15_H_10_O_4_	52.29	255.06484
23	Chrysophanol	Neg	C_15_H_10_O_4_	52.60	253.05070
24	Physcion	Neg	C_16_H_12_O_5_	54.54	283.06080

### 3.2 Effects of DF on the behavior of CCI rats

**Figure 2:**
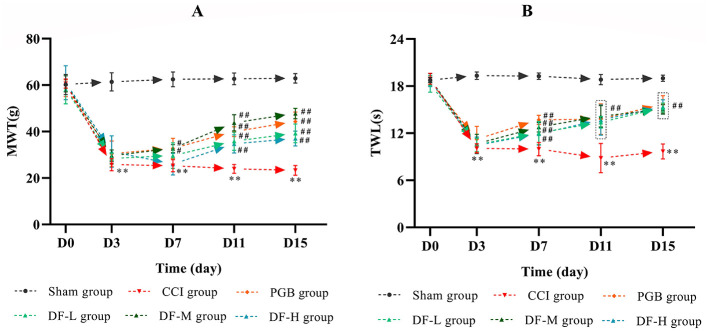
The effects of DF on the behavior in the CCI rats. **(A, B)** The MWT and TWL values among the sham, CCI, DF-L, DF-M, DF-H, and PGB groups before and 3, 7, 11, and 15 days after CCI surgery (*n* = 6 in each group). ^**^P < 0.01 compared with the sham group; ^#^*P* < 0.05 and ^*##*^*P* < 0.01 compared with the CCI group.

To assess the analgesic effect of DF, MWT and TWL were measured before and at 3, 7, 11, and 15 days post-CCI surgery (Figures 2A, B). There were no significant differences in MWT and TWL values across the groups prior to surgery (*P* > 0.05). From the third day after operation, y the third postoperative day, MWT and TWL values in the CCI group significantly declined and remained low compared to the sham group (*P* < 0.01). Starting from day 7 post-drug administration, both the DF-M and pregabalin groups exhibited a significant increase in MWT and TWL compared to the CCI group (*P* < 0.05). The DF-L and DF-H groups also demonstrated varying improvements in these values during the treatment period. These findings indicate that DF has a notable analgesic effect in CCI rats.

### 3.3 Identification of targets of DF against NeP

**Figure 3:**
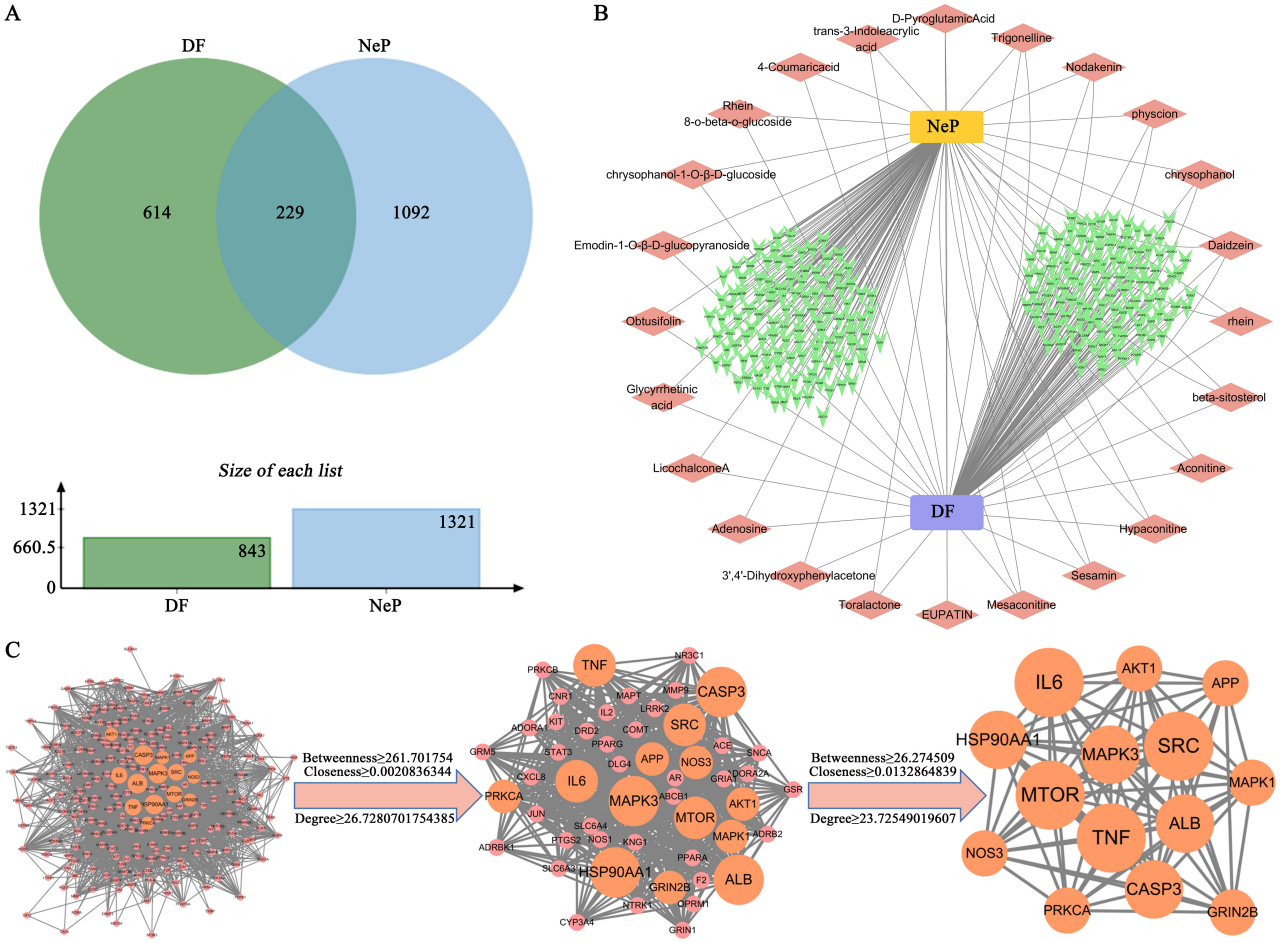
Prediction of the targets of DF against NeP by network pharmacology. **(A)** The Venn diagram of the common targets of DF and NeP. **(B)** Drug-disease-ingredients-targets network. **(C)** PPI network of targets of DF against NeP.

Network pharmacology was utilized to predict the targets of DF against NeP. The 24 compounds of DF identified by UHPLC-HRMS served as input for the analysis. This approach yielded 843 targets linked to the 24 DF ingredients and 1,321 targets associated with NeP (Figure 3A). Among them, 229 targets were identified as common targets between DF and NeP (Figure 3A). A drug-disease-ingredients-targets network was constructed, as shown in Figure 3B. To explore the interactions among DF's targets in relation to NeP, a protein-protein interaction (PPI) network was created using STRING. This network included 228 nodes and 3,047 edges, with an average degree of 26.7 (Figure 3C). Each node represented a target, with node size reflecting degree value and connection strength. Topological analysis of the PPI network was performed to identify core targets, revealing 51 targets with values exceeding the 2-fold median (Figure 3C). Further analysis in STRING, using the 2-fold median as the criterion, identified the top 14 targets, which included TNF, MTOR, MAPK3, SRC, ALB, CASP3, AKT1, APP, MAPK1, GRIN2B, PRKCA, NOS3, HSP90A1, and IL6 (Figure 3C). These 14 targets were considered the core targets of DF in the treatment of NeP.

### 3.4 GO and KEGG pathway enrichment analysis of the targets of DF against NeP

**Figure 4:**
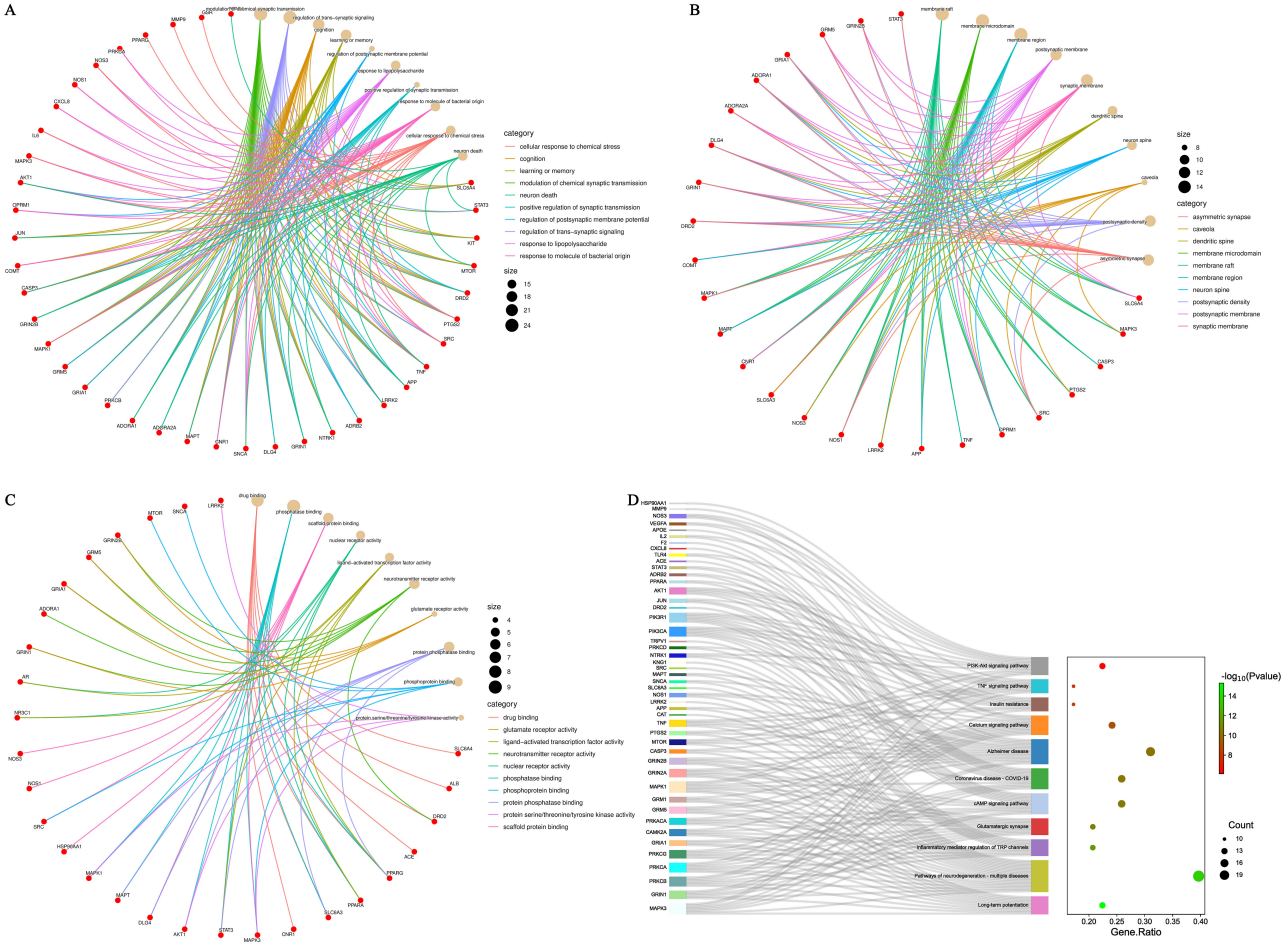
GO and KEGG enrichment analysis of the core targets of DF against NeP. **(A)** GO enrichment analysis of BP. **(B)** GO enrichment analysis of CC. **(C)** GO enrichment analysis of MF. **(D)** KEGG enrichment analysis.

GO and KEGG enrichment analyses were conducted using the Metascape database for the 51 targets of DF against NeP. GO enrichment indicated that these targets are involved in processes such as modulation of chemical synaptic transmission, neuron death and positive regulation of synaptic transmission in biological process (BP); asymmetric synapse, neuron spine and synaptic membrane in cellular component (CC); and neurotransmitter receptor activity, phosphoprotein binding and protein serine/threonine/tyrosine kinase activity in molecular function (MF; Figures 4A–C). KEGG analysis revealed that the 51 targets were primarily associated with pathways such as the PI3K-AKT signaling pathway, TNF signaling pathway, calcium signaling pathway, glutamatergic synapse, inflammatory mediator regulation of TRP channels and pathways of neurodegeneration-multiple diseases (Figure 4D), all of which were closely related to NeP. Notably, the PI3K-AKT and TNF signaling pathways showed the most significant enrichment (Figure 4D), suggesting their potential central roles in DF's mechanisms of action.

### 3.5 DF treatment reduced inflammatory cytokines levels in CCI model spinal cords

**Figure 5:**
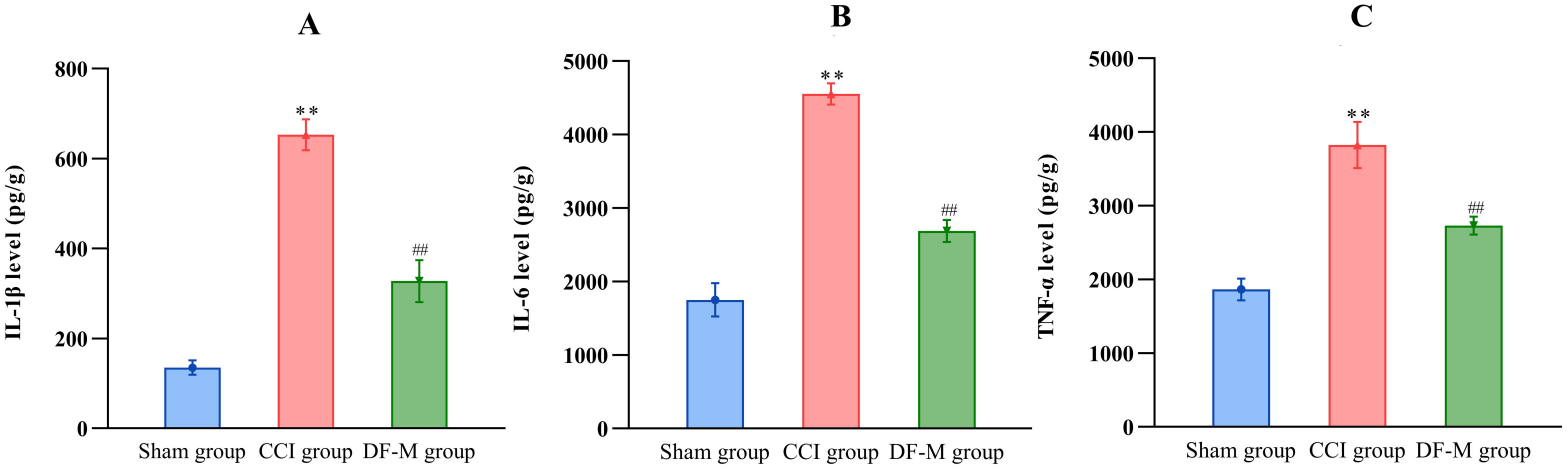
The effects of DF on the levels of inflammatory cytokines in the spinal cord of the CCI rats. **(A–C)** The levels of IL-1β, IL-6, and TNF-α in the spinal cord of the sham, CCI and DF-M group (*n* = 3 in each group). **P < 0.01 compared with the sham group; ^##^*P* < 0.01 compared with the CCI group.

To investigate the anti-inflammatory effects of DF, levels of IL-1β, IL-6, and TNF-α in the spinal cord were assessed using ELISA. The medium-dose DF granules were selected due to their significant impact on hyperalgesia following CCI surgery. Results indicated that DF significantly reduced the elevated levels of IL-1β, IL-6, and TNF-α in the spinal cord caused by CCI surgery (*P* < 0.01), highlighting DF's inhibitory effects on neuroinflammation in NeP (Figures 5A–C).

### 3.6 DF treatment suppressed TNF-α and PI3K-AKT signaling pathways in in CCI model spinal cords

**Figure 6:**
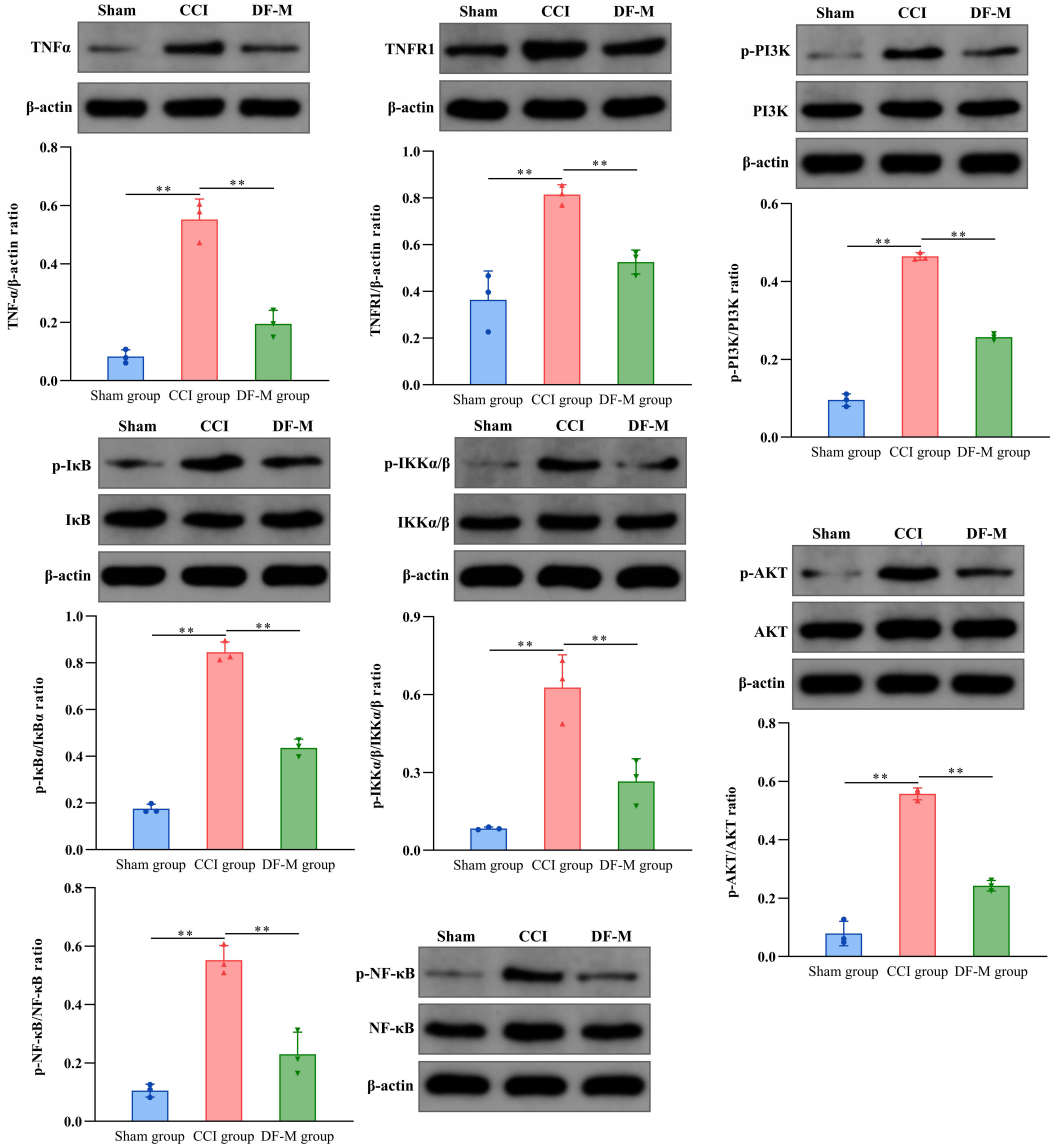
Effects of DF on the expressions of TNF-α and TNFR1, as well as the phosphorylation of PI3K, AKT, IKKα/β, IKBα, and NF-κB in the spinal cord of the sham group, CCI group, and DF-M group. Data are expressed as the mean ± SD (*n* = 3). **p < 0.01 compared to the CCI group.

**Figure 7:**
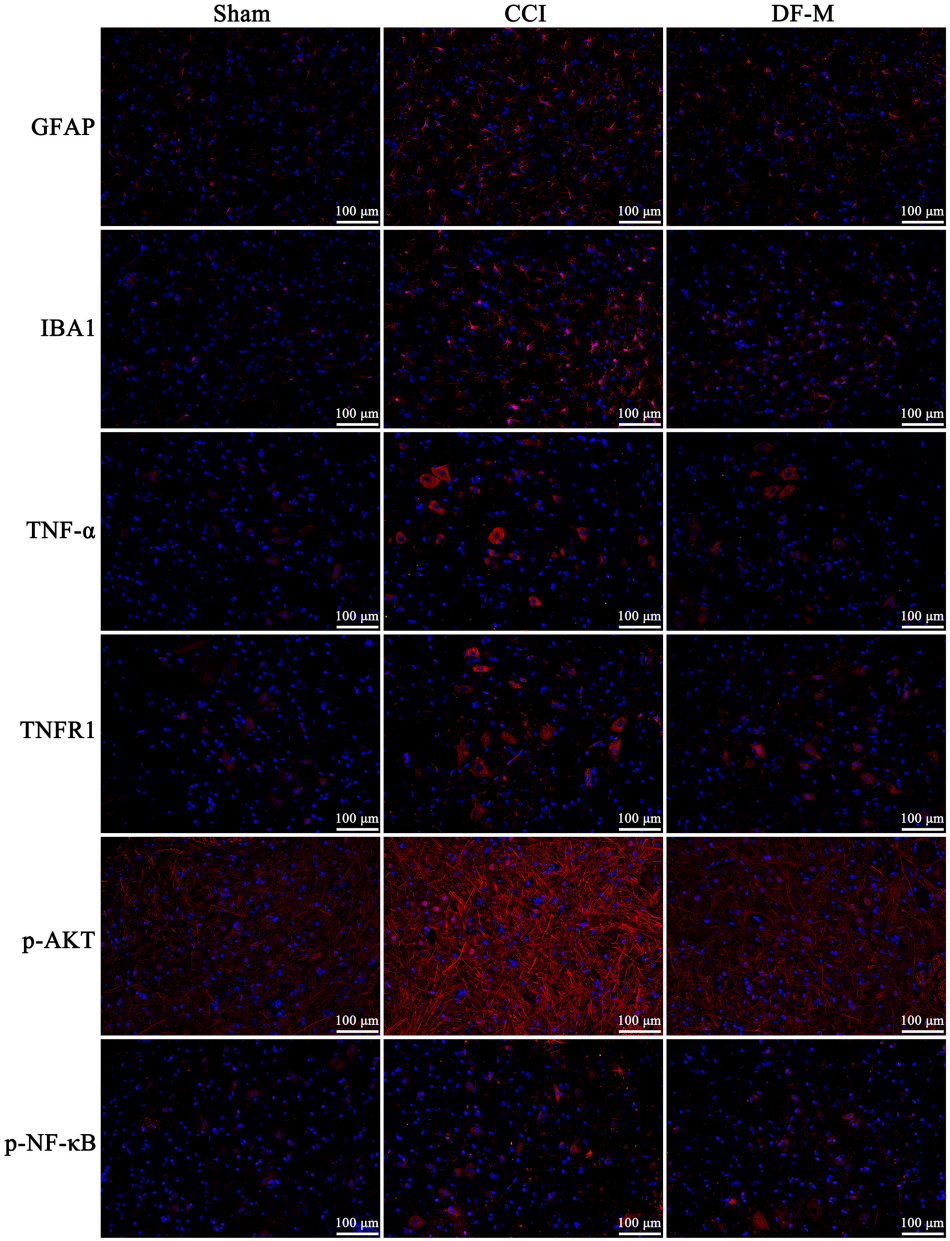
Immunofluorescence detection of the expressions of GFAP, IBA1, TNF-α, and TNFR1, as well as the phosphorylation of AKT and NF-κB in the spinal cord of the sham group, CCI group, and DF-M group. Spinal cord sections were stained with DAPI to visualize the nuclei (blue), while antibodies targeting the specific proteins were used for immunostaining (red).

Based on KEGG enrichment analysis and inflammatory cytokine measurements, it was clear that the TNF-α and PI3K-AKT signaling pathways played crucial roles in DF's mechanism for treating NeP. To further examine this, the expression levels of key molecules involved in these pathway were evaluated in the spinal cord tissue. Results showed elevated levels of TNF-α, TNFR1, and phosphorylated PI3K, AKT, IKKα/β, IKBα, and NF-κB in the model group compared to the sham group (*P* < 0.01, Figure 6). In contrast, the DF-M group exhibited lower levels of TNF-α, TNFR1, and phosphorylated proteins compared to the model group (*P* < 0.01, Figure 6). These findings suggested that DF effectively suppressed the TNF-α and PI3K-AKT signaling pathway in CCI rat spinal cords. Additionally, immunofluorescence results indicated that expressions of the astrocyte marker GFAP, the microglia marker IBA, TNF-α, TNFR1, and phosphorylated AKT and NF-κB were significantly elevated in the model group relative to the sham group, while levels in the DF-M group were markedly reduced compared to the model group (Figure 7). Collectively, these results demonstrate that DF mediates its analgesic effects through the inhibition of the TNF-α and PI3K-AKT signaling pathways.

### 3.7 The molecular docking verification

**Figure 8:**
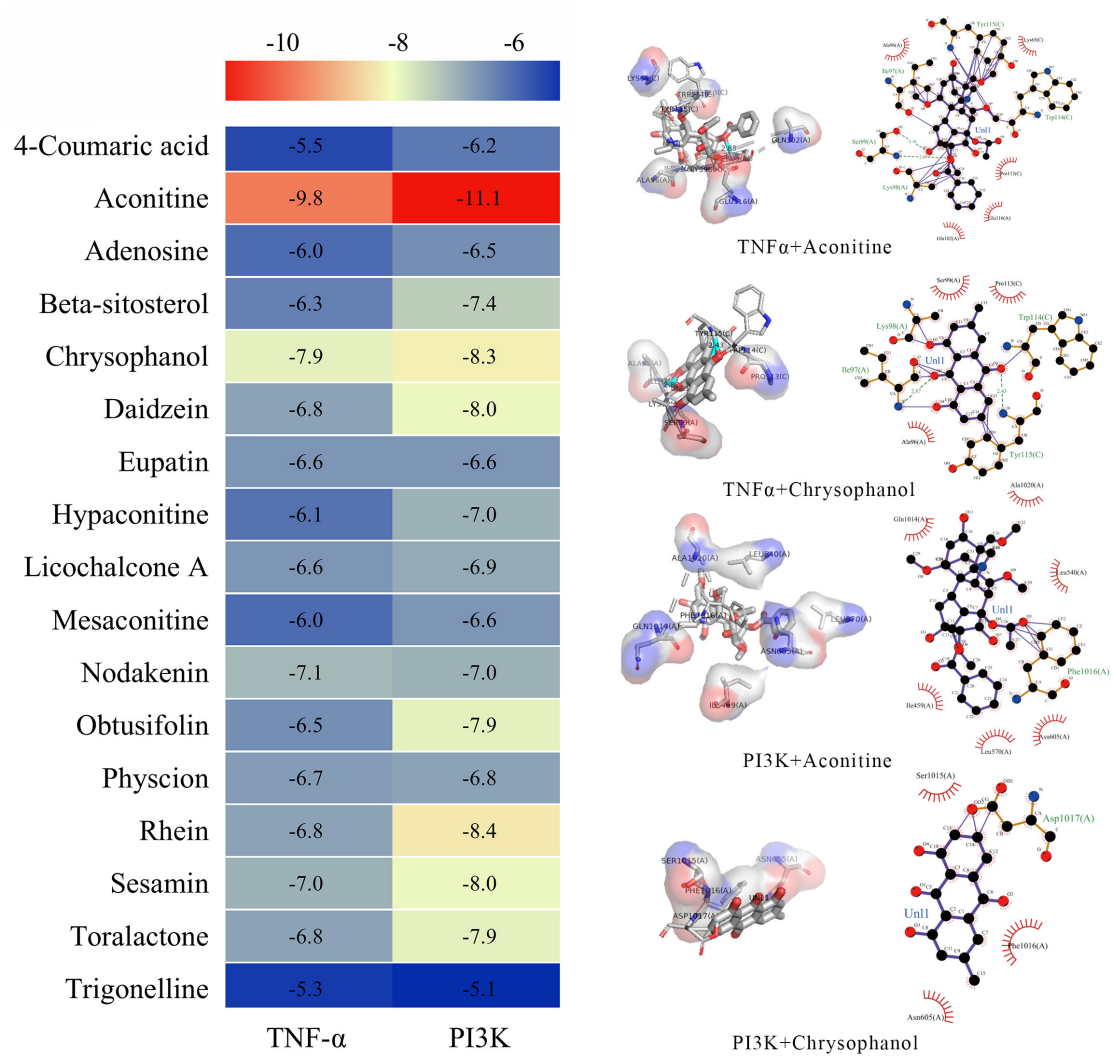
Molecular docking analysis of the binding affinity between the ingredients of DF and PI3K and TNF-α.

To further investigate the direct interactions between DF ingredients and TNF-α, PI3K, and AKT, molecular docking was performed to predict their binding affinities. The results indicated that aconitine, chrysophanol, rhein, sesamin, and toralactone exhibited strong binding capabilities with both TNF-α and PI3K (Figure 8). Nodakenin, daidzein, and physcion showed significant binding affinity with TNF-α, while daidzein, obtusifolin, and beta-sitosterol demonstrated good binding potential with PI3K (Figure 8). These findings suggest that the diverse ingredients of DF can directly interact with specific targets, indicating that individual DF components may engage multiple targets associated with NeP.

## 4 Discussion

NeP is marked by a variety of clinical manifestations and a complex pathogenesis, significantly affecting patients' quality of life and imposing considerable economic, physical, and psychological burdens (Blyth, 2018). Therefore, it is crucial to urgently explore effective and safe treatments that address the underlying mechanisms of NeP. Recently, several TCM formulations have shown promise in alleviating NeP through multi-target strategies (Zhang et al., 2014, 2021). DF is a notable analgesic candidate due to its warming and pain-relieving properties, along with its ability to suppress inflammatory responses (Tu et al., 2014; Guo et al., 2022). Central sensitization is a key mechanism underlying the development and maintenance of neuropathic pain, significantly amplifying pain signaling within the nervous system. The spinal cord is the primary site where this process occurs, integrating and modulating nociceptive input, making it a crucial focus for understanding the persistence of neuropathic pain.

This study aims to elucidate, for the first time, the pharmacological mechanisms responsible for the analgesic effects of DF by integrating network pharmacology with experimental validation.

In our study, we first identified the components of DF using UHPLC-HRMS, successfully detecting a total of 24 distinct ingredients. Several of these compounds have been reported to possess anti-inflammatory, analgesic, and neuroprotective properties. Notably, aconitine, daidzein, licochalcone A, mesaconitine, and obtusifolin have demonstrated significant analgesic effects in various acute and chronic pain models. These effects are mediated through the regulation of transient receptor potential (TRP) channels, inhibition of microglial activation and neuroinflammation, as well as the suppression of neuronal apoptosis (He et al., 2014; Sun et al., 2020; Cankal et al., 2021; Deng et al., 2021; Jin et al., 2023; Zafar et al., 2023). Additionally, sesamin has been shown to provide significant pain relief in patients with rheumatoid arthritis and to improve pain-related behaviors in rat models by reducing inflammatory mediators and inhibiting ROS-induced apoptosis (Deng et al., 2018; Helli et al., 2019). Furthermore, trigonelline, eupatin, and physcion have exhibited neuroprotective effects across various neurological disorders (Xunli et al., 2019; Chou et al., 2020; Liang et al., 2023).

We found that DF exhibited a significant analgesic effect in CCI rats and reduced inflammatory cytokine levels in the spinal cord. Neuroinflammation, defined as localized inflammation within the nervous system, is a key factor in the development of NeP. This condition is characterized by increased vascular permeability, the migration of inflammatory cells, activation of glial cells, and heightened secretion of inflammatory mediators (Teixeira-Santos et al., 2020). The interaction between microglia and astrocytes plays a crucial role in neuroinflammation associated with NeP. In our study, DF treatment resulted in significantly lower levels of IL-1β, IL-6, and TNF-α in the spinal cord compared to the CCI models, suggesting that DF effectively inhibits the CCI-induced inflammatory response. Additionally, we observed a marked reduction in the abnormal elevation of ionized calcium-binding adaptor molecule 1 (IBA1) and glial fibrillary acidic protein (GFAP) in response to DF treatment. These proteins are widely recognized markers for reactive microglia and astrocytes in the central nervous system (Chen et al., 2022; Hiraga et al., 2022).

To explore the mechanism by which DF alleviates neuroinflammation in the treatment of NeP, we performed network pharmacology analysis. The findings indicated that DF targets were primarily enriched in inflammation-related pathways, including the TNF-α signaling pathway, PI3K-AKT signaling pathway, and NF-κB signaling pathway. TNF-α, a key pro-inflammatory cytokine released from activated microglia, initiates a cytokine storm and activates pain-related pathways through TNF receptors, thus influencing central sensitization (Zhao et al., 2017; Chen et al., 2018). Numerous studies have shown that inhibiting TNF-α or genetically knocking out TNFR1 prevents NeP-induced hyperalgesia and aberrant synaptic plasticity changes (Liu et al., 2017; Lamacchia et al., 2019; Son et al., 2022). The transcription factor nuclear factor κB (NF-κB), a downstream effector of the TNF-α signaling pathway, acts as a master regulator of inflammation and other pathological processes in NeP (Niederberger and Geisslinger, 2008; Han et al., 2023). When TNF-α binds to TNFR1, the IκB kinase (IKK) complex, comprising IKKα, IKKβ, and the regulatory subunit IKKγ, is recruited and activated. This activated IKK phosphorylates NF-κB inhibitor-α (IκBα), resulting in its polyubiquitination and subsequent proteasomal degradation (Varfolomeev and Vucic, 2018). Consequently, NF-κB is released and translocates to the nucleus, where it activates various target genes and stimulates the production and release of inflammatory cytokines (Ding and Chen, 2023). Several studies have reported elevated expression of NF-κB in the spinal cords of various NeP animal models, and inhibiting NF-κB activity significantly reduces both the inflammatory response and neuronal excitability (Miao et al., 2020; Chen et al., 2023a,b; Khan et al., 2023). In our study, DF-M treatment significantly reduced the expression of TNF-α and TNFR1, as well as the phosphorylation levels of IKKα/β, IκBα, and NF-κB in the spinal cord of CCI rats. Thus, we conclude that the inhibition of TNF-α signaling is a critical mechanism underlying the analgesic effects of DF.

PI3K, a lipid kinase, plays a crucial role in several key intracellular signaling processes and regulates major cellular functions through its downstream target, AKT (He et al., 2022). Previous research has shown that the PI3K-AKT signaling pathway is involved in various pathological mechanisms associated with the onset and persistence of NeP by phosphorylating multiple critical downstream molecules, including NF-κB (Chen et al., 2017; Liu et al., 2018; Ji and Xu, 2021). In our study, we noted a significant reduction in the phosphorylation levels of PI3K and AKT in the spinal cords of rats treated with DF-M compared to CCI rats. These results indicate that DF may exert its analgesic effects in NeP by inhibiting the PI3K-AKT signaling pathway. However, we acknowledge that the mechanisms of action of DF are complex and may involve multiple signaling pathways. In future studies, we will employ multi-omics analysis and other methods to conduct in-depth investigations into other enriched signaling pathways, aiming to comprehensively elucidate the mechanisms of action of DF against NeP.

## 5 Conclusion

In conclusion, our findings indicate that DF effectively reduces CCI-induced mechanical and thermal hyperalgesia by inhibiting neuroinflammation in the spinal cord, primarily mediated through the TNF-α and PI3K-AKT signaling pathways. These results offer a new perspective for research aimed at developing analgesic drugs that target neuroinflammation.

## Data Availability

The raw data supporting the conclusions of this article will be made available by the authors, without undue reservation.
